# Nodular image in the appendix observed on ultrasound: endometriosis or neuroendocrine neoplasia?

**DOI:** 10.61622/rbgo/2024AO01

**Published:** 2024-03-15

**Authors:** Raphael Federicci Haddad, Bruna Cestari de Azevedo, Leandro Accardo de Mattos, Sergio Podgaec

**Affiliations:** 1 Hospital Israelita Albert Einstein São Paulo SP Brazil Hospital Israelita Albert Einstein, São Paulo, SP, Brazil.; 2 Universidade de São Paulo Faculdade de Medicina Departamento de Obstetrícia e Ginecologia São Paulo SP Brazil Departamento de Obstetrícia e Ginecologia, Faculdade de Medicina, Universidade de São Paulo, São Paulo, SP, Brazil.

**Keywords:** Endometriosis, Carcinoid tumor, Carcinoma, neuroendocrine, Appendix, Ultrasonography

## Abstract

**Objective:**

To evaluate the association between clinical and imaging with surgical and pathological findings in patients with suspected neuroendocrine tumor of appendix and/or appendix endometriosis.

**Methods:**

Retrospective descriptive study conducted at the Teaching and Research Institute of *Hospital Israelita Albert Einstein*, in which medical records and databases of patients with suspected neuroendocrine tumor of appendix and/or endometriosis of appendix were analyzed by imaging.

**Results:**

Twenty-eight patients were included, all of which had some type of appendix alteration on the ultrasound examination. The pathological outcome of the appendix found 25 (89.3%) lesions compatible with endometriosis and three (10.7%) neuroendocrine tumors. The clinical findings of imaging and surgery were compared with the result of pathological anatomy by means of relative frequency.

**Conclusion:**

It was possible to observe a higher prevalence of appendix endometriosis when the patient presented more intense pain symptoms. The image observed on ultrasound obtained a high positive predictive value for appendicular endometriosis.

## Introduction

Endometriosis is a common inflammatory disease that affects about 10% of women of reproductive age.^(1)^ Among the patients with pain and infertility, 10 to 20% have deep endometriosis, a more severe form of the condition, histologically defined by lesions that extend by 5 mm or more in the peritoneum.^(2)^ In general, this form is responsible for more intense symptoms and to detect these lesions imaging methods are used, such as magnetic resonance imaging and pelvic and transvaginal ultrasound (US) with bowel preparation.^(3-6)^

One of the sites that can be affected in deep endometriosis is the appendix, present in less than 1% of all cases of pelvic endometriosis and approximately 3% of gastrointestinal endometriosis.^(7,8)^ The clinical condition of these patients may be nonspecific with a complaint of chronic pelvic pain; pain in the right iliac fossa, mimicking acute appendicitis; presenting gastrointestinal bleeding, intussusception, obstruction or intestinal perforation; and may be asymptomatic.^(7,9,10)^ There is also a description in the literature of patients with unusual presentation of cyclic epigastric pain.^(11)^

The definitive diagnosis is established with the histopathological analysis of the appendix removed by surgery.^(1,9)^ Other tests such as CA125 dosage and computed tomography were not sufficient to improve preoperative evaluation.^(9,10)^

The importance of the present study lies in the differential diagnosis of appendix tumors, of which the most frequent is neuroendocrine.^(11,12)^ This is because this appendicular tumor has a clinical presentation similar to that of appendicular endometriosis.^(11,13)^ Its diagnosis is also definitively performed with histopathological examination, but imaging tests such as US, magnetic resonance imaging and computed tomography can be used.^(14)^ Colonoscopy, although not efficient for diagnosing the appendix tumor, has its indication in the search for synchronous neoplasms in the gastrointestinal tract.^(15,16)^

Thus, the differentiation between endometriosis and neuroendocrine tumor of appendix remains a challenge today. Therefore, the aim of this study was to evaluate the association between clinical condition and imaging with surgical findings in patients with suspected neuroendocrine tumor and/or appendix endometriosis.

## Methods

This is a retrospective descriptive study conducted at the Institute of Teaching and Research of the *Hospital Israelita Albert Einstei*n, where medical records and databases of patients with suspected neuroendocrine tumor and/or appendix endometriosis between 2014 and 2020 were analyzed in US findings.

In the period described above, all patients seen by a single gynecologist (SP) with suspected endometriosis were referred for pelvic and transvaginal US with intestinal preparation for mapping the disease performed by a single radiologist (LAM), with more than 15 years of experience in this type of examination. All patients who presented alterations in the appendix underwent surgical treatment and were included in the present study.

Clinical, US, surgical and pathological data of all patients were analyzed. Regarding clinical data, quantitative (age, weight (in kg), height (in meters), BMI (body mass index), number of pregnancies and visual analog pain scale related to pain complaints with scores ranging from 0 to 10) and qualitative data (ethnic group, presence of infertility, depth dyspareunia, dysmenorrhea, chronic pelvic pain, cyclic bowel changes, cyclic urinary changes and changes in pelvic physical examination) were collected.

Regarding data from US examinations and surgeries, qualitative data (adenomyosis, presence of lesions in the peritoneum, bladder, retrocervical, paracervical, uterosacral ligaments, vagina, ureter, ovary, sigmoid rectum, ileum, cecum, appendix and diaphragm) and quantitative (size of the lesions) were analyzed.

The sample was described from the mean and standard deviation, minimum and maximum, median and quartiles for quantitative variables and by the absolute and relative frequencies for qualitative variables. Data normality was verified from the Shapiro-Wilk test, boxplot graphs, histograms and quartile comparation plots.^(17)^ The analyses were carried out from the statistical package Statistical Package for the Social Science - SPSS, v.26.0.^(18)^

The study was approved by the ethics committee of the institution 5251518 (CAAE: 39128720.3.0000.0071).

## Results

We included 28 patients who had undergone pelvic and transvaginal US with intestinal preparation and who showed some alteration in the appendix, including suspected endometriosis or neuroendocrine tumor. The studied group had a mean age of 36.9 years with a standard deviation of 3.9 years, ranging from 29.6 to 45.9 years, with 27 patients (96.4%) being white. The pathological outcome of the appendix found 25 (89.3%) lesions compatible with endometriosis and three (10.7%) neuroendocrine tumors. Of the 28 patients, 11 (39.3%) had already undergone surgical treatment of endometriosis. Regarding the clinical condition, the main complaints were dysmenorrhea (n=24, 85.7%), depth dyspareunia (n=9, 32.1%), pain at evacuation during the menstrual period (n=9, 32.1%), chronic pelvic pain (n=8, 28.6%) and infertility (n=5, 17.9%). Table 1 shows the other characteristics of the studied group and table 2, the frequency of symptoms by group (endometriosis and neuroendocrine tumor). In addition, as reported in table 3, 4 (16%) patients in the endometriosis group reported chronic pelvic pain of intensity greater than 7 on the visual analog pain scale (VAS) and 22 (88%) reported dysmenorrhea of intensity greater than 7. Meanwhile, 1 (33.3%) patient reported chronic pelvic pain and 2 (66.7%) reported dysmenorrhea, but none of intensity greater than 7 in VAS (Tables 2 and 3).

**Table 1:** Sample characteristics

Variables		n(%)	Average (SD)	Median (Q1-Q3)
Age		-(-)	36,9(2,9)	36,9 (33,6 - 40)
Ethical group				
	White	27(89)	- (-)	- (-)
	Black	1(3,6)	- (-)	- (-)
Marital status			- (-)	- (-)
	Single	3(10,7)	- (-)	- (-)
	Married woman	25(89,3)	- (-)	- (-)
Weight		-(-)	60,5(6,4)	59,3 (55,5 – 65)
	Height	-(-)	1,63(0,04)	1,62 (1,60 – 1,65)
	BMI	-(-)	22,82(2,15)	22,62 (20,88 – 24,5)

**Table 2:** Frequency of symptoms by group (endometriosis and neuroendocrine tumor)

Clinic	Endometriosis	Neuroendocrine tumor
	n(%)	n(%)
Chronic pelvic pain	7(28)	1(33)
Infertility	5(20)	0(0)
Depth dispareunia	8(32)	1(33)
Dysmenorrhoea	22(88)	2(67)
Abdominal pain	5(20)	0(0)
Cyclic bowel changes	13(52)	0(0)
Pain to evacuation	9(36)	0(0)
Cyclic urinary changes	2(8)	0(0)

**Table 3:** Comparison of symptom intensity according to visual analog pain scale by group (endometriosis and neuroendocrine tumor)

Clinic	Endometriosis			Neuroendocrine tumor		
	Average	Median	>7	Average	Median	>7
Chronic pelvic pain	7,71	9	4	6	6	0
Depth dispareunia	5,43	5	0	7	7	0
Dysmenorrhoea	8,23	8	22	7	5	0
Abdominal pain	8	7	2	-	-	-
Pain to evacuation	6,67	7	3	-	-	-

**Table 4:** Ultrasonographic findings by group (endometriosis and neuroendocrine tumor)

Region	Endometriosis		Neuroendocrine tumor	
	US	Surgery	US	Surgery
	n(%)	n(%)	n(%)	n(%)
Adenomyosis	2(8)	1(4)	1(33)	0(0)
Bladder	2(8)	2(8)	0(0)	0(0)
Peritoneum	-(-)	14(56)	-(-)	3(100)
Retrocervical	17(68)	20(80)	3(100)	2(67)
Vagina	2(8)	8(32)	1(33)	3(100)
Paracervical	2(8)	-(-)	0(0)	-(-)
Ureter	0(0)	4(16)	0(0)	1(33)
Ovary	8(32)	9(36)	3(100)	3(100)
Sigmoid rectum	10(40)	7(28)	2(67)	2(67)
Ileum	1(4)	1(4)	1(33)	0(0)
Cecum	3(12)	1(4)	0(0)	0(0)
Appendix	25(100)	25(100)	3(100)	3(100)
Thickening	4(16)	-(-)	1(33)	-(-)
Injury	19(76)	-(-)	1(33)	-(-)
Questioned	2(8)	-(-)	0(0)	-(-)
Mucocele	0(0)	-(-)	1(33)	-(-)

Table 4 describes the alterations observed on US examination and during surgery, subdivided according to the pathological result. We compared the clinical findings of imaging and surgery with the result of anatomic pathology. The comparison between the two groups was presented in the tables with the descriptions of frequencies. The presence of ovarian endometrioma and retrocervical nodule was observed only in patients in the endometriosis group, 4.3% and 39.1%, respectively.

In addition, two cases were observed in which the US finding was questioned. Both had as a result of the pathological study only appendicular endometriosis. When we include these lesions questioned, the PPV (positive predictive value) for endometriosis of the studied group found is 89.3%. When excluded, the PPV was 88.5%.

## Discussion

Endometriosis is a prevalent condition that generates discomfort and compromises the quality of life of patients.^(1,2)^ which may affect the intestine, which can cause chronic pelvic pain and intestinal symptoms, being one of the most severe forms and with more clinical repercussions of the disease.^(19)^ Within this group of patients, we found a higher rate of infertility, more chronic pain complaints and greater difficulty in treatment.^(20)^

One of the sites where the disease can be found is the appendix, which can be confused with appendicular neuroendocrine tumor injury.^(13,19)^ Therefore, its better diagnostic elucidation of the disease, provides less anxiety for the team and for the patient, better surgical planning, allows the selection of more specialized professionals for each case, multidisciplinary discussions and, consequently, a better therapeutic approach.^(6,19)^ For this better diagnostic understanding, specific imaging tests should be used that include pelvic and transvaginal US with intestinal preparation and pelvic magnetic resonance imaging, which play a fundamental role in better diagnostic elucidation and mapping of affected regions.^(5,21)^

Regarding these, they are complementary methods, but they present particularities that benefit the diagnosis according to the region studied. The first is more accurate for visceral structures of the pelvis such as the intestine and especially the ileum and appendix, due to better contrast resolution and peristalsis, which makes it difficult to visualize through magnetic resonance.^(4,22)^ The second allows a better visualization of the upper abdomen (diaphragm and hepatorenal space), of small ovarian lesions and in the evaluation of lesions that are far from the range of the US transducer, as in the case of the sacral plexus.^(6,21)^ In the present study, we selected patients who were submitted to US with intestinal preparation, performed by an experienced professional specialized in the condition in question, a fact that allowed greater standardization of the results and homogeneity of the same with the standard examination of gold in this investigation.

Moreover, as Savelli et al.^(23)^ demonstrated, the diagnostic accuracy of US in these cases is related to the physician's experience and the size of the nodule.^(22,23)^ Therefore, ultrasonography has high precision and is closely related to the sonographer's experience, which allows its use as a method of choice.^(22)^

The assertiveness of the identification of appendicular involvement by US in the study was high. In other words, in all cases that presented some US finding, the presence of lesion was confirmed (endometriosis or neuroendocrine tumor) and no false positive was identified. The US finding was questioned in only two of these cases and, as previously stated, when we included these lesions questioned, the positive predictive value (PPV) for endometriosis found was 89.3%. This confirms the high PPV of US examination for appendix lesions, especially when it comes to endometriosis.^(2)^ Thus, the US changes in the appendix should always be valued.

None of the patients studied who were diagnosed in the anatomopathological with neuroendocrine tumor presented carcinoid syndrome, which corroborates the rarity of this syndrome: only 5% of neuroendocrine tumors evolve to the carcinoid syndrome, a condition of greater severity.^(24-26)^

Regarding the pathological study of the appendix, it was not possible to perform comparative statistical analysis between the group with endometriosis and the neuroendocrine tumor group due to the number of cases with the latter diagnosis. However, when analyzing the relative and absolute frequencies of the parameters in relation to each group, a less exuberant clinical condition (lower intensity in the visual pain scale) was observed in cases of neuroendocrine tumor. This is in line with the fact that the neuroendocrine tumor is an indolent condition, presenting symptoms in the minority of cases.^(11,14)^

Symptoms reported by patients include deep dyspareunia, chronic pelvic pain, and dysmenorrhea. However, it is worth mentioning that these patients also presented involvement with endometriosis in other regions, which may be a bias in the analysis of this symptomatology. In the case of patients diagnosed with endometriosis on pathological examination of the appendix, they reported the same symptoms with a higher intensity in the majority and added to infertility, abdominal pain, cyclic bowel and urinary changes and pain on evacuation. Perhaps this points to a relationship of appendicular injury due to endometriosis with a more extensive involvement of the disease.

Another interesting finding was the higher incidence of endometriosis when compared to appendicular neuroendocrine tumor, as reported in the literature.^(27,28)^ This is in line with the fact that this type of tumor is rare, with its incidence ranging from 0.3 to 0.9%, and its diagnosis is a finding in the investigation of other diseases, such as endometriosis itself, in the vast majority.^(24,26,29)^

Moreover, in our sample, the only case with the presence of mucocele in an US report was diagnosed with neuroendocrine tumor. However, no features were observed on US that directed to the diagnosis, a fact that is corroborated by the literature.^(11)^

The age group found in both groups was similar and is in accordance with the literature. Although its sequelae can be found in postmenopausal women, most cases of endometriosis are found in menacme patients.^(29)^ In the postmenopausal period, gastrointestinal symptoms may be the only manifestations.^(4)^ Neuroendocrine tumors affect patients in an age group similar to patients with endometriosis and have also similar symptoms, with rare but predominantly intestinal repercussions. As limitations of the study, we highlight the fact that it is a retrospective study based on analysis of medical records and the small number of cases with neuroendocrine tumor. Positive points are the large number of cases with appendicular endometriosis and the fact that all US examinations were performed by the same professional.

It is worth emphasizing the importance of preoperative imaging as a form of endometriosis mapping and better surgical planning. This accurate preoperative knowledge of the presence and extent of possible lesions helps in the planning of appropriate treatment, in advising women on the risks and complications of surgery and in deciding on a possible surgical treatment.^(23)^ Therefore, it was possible to confirm the need for appendectomy for the cases indicated by US. Laparoscopy is the route of choice for cases of identification of these macroscopic alterations of the appendix in pre-surgical imaging. If any incidental alteration scans are found in the intraoperative appendix, appendectomy should also be performed.^(5)^

## Conclusion

A higher prevalence of appendix endometriosis was observed when the patient presented pain symptoms more intensely according to the visual pain scale. This may point to a higher probability of appendix endometriosis in cases of more exuberant endometriosis clinic, associated with US finding of appendicular injury. However, prospective studies are necessary to confirm such observation. In addition, US was shown to be an examination with a high positive predictive value for endometriosis lesions and even on pathological examination in which endometriosis was not characterized, neuroendocrine tumor was described in all cases. That is, there was no false positive for lesions in the appendix identified on US.
